# Spatial and temporal sex ratio bias and *Wolbachia*-infection in New Zealand Crambidae (Lepidoptera: Pyraloidea)

**DOI:** 10.3897/BDJ.8.e52621

**Published:** 2020-07-07

**Authors:** Renate Wöger, Roland Wöger, Matthias Nuss

**Affiliations:** 1 Senckenberg Museum of Zoology, Dresden, Germany Senckenberg Museum of Zoology Dresden Germany

**Keywords:** Pyraloidea, Crambidae, Wolbachia-infection, sex ratio bias, New Zealand

## Abstract

The New Zealand fauna of snout moths (Pyraloidea) predominantly consists of endemic species. During 2017 and 2018, 56 species of Pyraloidea in 1,749 individuals were collected at 14 localities. All species were screened for *Wolbachia*-infection, with specimens of eight species (14%) being positive, of which six species belong to Scopariinae. This is the first record of *Wolbachia*-infection amongst New Zealand Lepidoptera. The most common pyraloid species, *Eudonia submarginalis* and *Orocrambus flexuosellus*, were analysed for a larger set of individuals looking for sex ratio and *Wolbachia*-infection. There is a sex ratio bias towards females in both species, but it varies in space and time. *Wolbachia* is found in all populations of *E. submarginalis* with 10–80% of the tested individuals being positive, depending on locality. No *Wolbachia*-infection has been found in *O. flexuosellus*. Thus, sex ratio bias might be linked to *Wolbachia*-infection in *E. submarginalis*, but not in *O. flexuosellus*.

## Introduction

Snout moths (Pyraloidea) are one of the megadiverse subgroups of Lepidoptera, occurring worldwide with more than 16,000 described species (Nuss et al. 2020.) Its 19 phylogenetically-supported subfamilies Regier et al. 2012, Mally et al. 2019, Leger et al. 2019) display a great morphological and ecological diversity. The larvae are phytophagous, feeding on most groups of plants, but they could also be detritivorous, congrophagus, predative and parasitive. The majority of larvae are terrestrial, but one lineage is adapted to aquatic habitats. In New Zealand, there are 250 described species of Pyraloidea, of which most are endemic besides 11 species which have been introduced by humans and a few also occurring in Australia. Compared to the world fauna, there is a strong bias amongst the endemics towards Scopariinae (129 species) and Crambinae (81 species) (Nuss et al. 2020). Larvae of most scopariine species are feeding on Bryophyta, those of Crambinae on Poales (Leger et al. 2019). The taxonomic inventory of pyraloids in New Zealand was largely completed by the 1970s (Nuss et al. 2020). Besides the faunistic work by Patrick (2014) and papers by Hoare (2001), Hoare (2011) also considering pyraloids, there is little recent research attention on Pyraloidea from New Zealand. Hence, we surveyed pyraloids in New Zealand during 2017 and 2018. Subsequently, moths were identified using wing pattern and genitalia structures and we started to establish a barcode library for New Zealand Pyraloidea (Wöger et al. in prep). During this work, a sex ratio bias in two of the most common pyraloid species, *Eudonia submarginalis* and *Orocrambus flexuosellus* has been recognised.

A sex ratio bias can be caused by different factors. One is a sexual dimorphism in timing of emergence (Degen et al. 2015). This phenomenon is often called “protandry” for the emergence of males before females within one population (Fagerström and Wiklund 1982, Bulmer 1983, Holzapfel and Bradshaw 2002, Degen et al. 2015) and “protogyny” when females emerge before males (Buck 2001, Degen et al. 2015). There are several hypotheses explaining these phenomena (e.g. Fagerström and Wiklund 1982, Wiklund and Solbreck 1982, Wang et al. 1990, Wedell 1992, Morbey and Ydenberg 2001, Holzapfel and Bradshaw 2002, Larsen et al. 2012, Degen et al. 2015) .

A sex ratio bias can be also caused by *Wolbachia*-infection (Jiggins et al. 2001, Werren et al. 2008). *Wolbachia* bacteria (Alphaproteobacteria) are common and widespread in reproductive tissues of arthropods (O'Neill et al. 1992, Werren et al. 2008). A *Wolbachia*-infection may cause induction of cytoplasmatic incompatibility (Stouthamer et al. 1993, Sasaki et al. 2002, Werren et al. 2008), parthenogenesis (Arakaki et al. 2001, Dyson et al. 2002), feminisation (Werren et al. 2008) and “male killing” (Jiggins et al. 2001), the latter three resulting in a sex ratio bias. Some *Wolbachia* strains are multi-potent and able to induce more than one mode of changing the sex ratio (Hurst et al. 2000, Werren et al. 2008). However, not all strains change the sex ratio of their host, some are commensal or even mutualistic (Hosokawa et al. 2009, Hamm et al. 2014, Newton and Rice 2020).

Based on molecular data (multi-locus sequence typing), 16 supergroups of W*olbachia* (A-Q) are currently recognised (Baldo and Werren 2007, Ros et al. 2009, Gerth et al. 2014, Glowska et al. 2015). The genetic variability amongst the supergroups is interpreted in favour of the existence of more than one species (Ellegaard et al. 2013, Ramirez-Puebla et al. 2015), but there is dispute about it (Lindsey et al. 2016).

*Wolbachia*-infection rates vary inter- and intraspecifically (e.g. Hilgenboecker et al. 2008, Werren et al. 2008, Zug and Hammerstein 2012) and are affected by geographical circumstances and the host’s fitness (Unckless et al. 2009, Ahmed et al. 2015).

*Wolbachia* is typically transmitted maternally through the cytoplasm of the eggs (Zug and Hammerstein 2012), with effects to the mitochondrial genetic structure of their host species (Jiggins 2003, Narita et al. 2009, Kodandaramaiah et al. 2013). The occurrence of identical *Wolbachia* strains in different host species suggests horizontal transmission (Ahmed et al. 2016, Chrostek et al. 2017, Ilinsky and Kosterin 2017, Paniagua Voirol et al. 2018), even though the mechanism behind is largely unknown.

Though there is a comprehensive bibliography about *Wolbachia*, there are still gaps in surveying *Wolbachia* amongst taxa and regions. For New Zealand, it has been first recorded just recently from Orthoptera, Psocoptera, Diptera and Hymenoptera (Bridgeman et al. 2018), but there are still no records from Lepidoptera. Since we found some sex ratio bias towards females in two of the most common pyraloid species in New Zealand, we took this as the occasion to test the material from our survey for *Wolbachia*-infection.

## Materials and methods

### Fieldwork

A survey of Pyraloidea in New Zealand has been undertaken during January and February of the years 2017 and 2018. A total of 56 species in 1,749 specimens were collected both during the day and also attracted to artificial UV light for 3–4 hours after nightfall. Collecting localities were visited one to six times, depending on travel logistics and weather conditions. The moths were collected from 14 localities, three of them being situated in Taranaki (North Island) and 11 localities scattered over the South Island. At each locality, all pyraloid individuals attracted by the UV light were collected. Specimens were killed using cyanide or ethyl acetate, pinned and dried for transportation. After fieldwork, moths were labelled and sorted to morpho-species. Specimens were identified by the authors using the database of the Landcare research Auckland (landcareresearch.co.nz) (Hoare 2020) and revision of the genus Orocrambus (Gaskin 1975). These resources are based on external morphology and genitalia dissection.

Nomenclature and taxonomy are based on the Global Information System on Pyraloidea (GlobIZ) (Nuss et al. 2020). In cases when wing pattern elements were not sufficient for species identification, genitalia dissections were made, following the protocol by Robinson (1976) and Nuss (2005).

### Sex ratio

The sex ratio was identified in the two most commonly collected species *Eudonia submarginalis* and *Orocrambus flexuosellus*. To distinguish males and females, we dissected the abdomen. The dissection followed Robinson (1976) and Nuss (2005) and analysis of morphological structures of genitalia was carried out using a stereomicroscope Euromex NexiusZoom NZ.

Record data of *E. submarginalis* and *O. flexuosellus* were separated into location and year. If we visited a locality more than once, the collected individuals were pooled. We tested for a significant departure from a 1:1 sex ratio by chi-square-tests using SPSS (Statistical Package for Social Science, IBM®) at all localities where more than 15 individuals were collected.

Phenograms are generated using Brian Patrick’s records from iNaturalist (iNaturalist.org) (Patrick 2014) together with the data from our surveys in 2017 and 2018.

### DNA extraction, PCR and sequencing

Genomic DNA was extracted from dried abdomens using the *Genomic DNA from tissue* kit (Macherey-Nagel, Düren, Germany), following the manufacturer‘s standard protocol for animal tissue.

PCR was performed to amplify the mitochondrial cytochrome oxidase I gene (COI) from the extracted DNA using the primer pair HybHCO/HybLCO. These primers contain a universal primer tail (T7), which is also used for sequencing (Wahlberg and Wheat 2008). The PCR was performed in 20 µl reactions, containing 10 pmol of each primer, 10mM dNTPs, 2 µl PCR 10x OptiBuffer, 100mM MgCl_2_ and 0.5 U taq DNA Polymerase (BIORON GmbH Ludwigshafen). After an initial phase at 95ºC for 5 min, the temperature profile was 95ºC for 30 sec, 50ºC for 30 sec and 72ºC for 45 sec for a total of 38 cycles. The final elongation temperature was 72ºC for 10 minutes followed by a cooling phase at 8ºC.

To determine amplicon presence and size, we examined PCR results via gel electrophoresis on a 1% agarose gel and GelRed as dye agent.

The samples with successful PCR were sequenced and tested for presence of *Wolbachia* DNA. If the COI Barcode PCR failed, we excluded the sample. For sequencing work, we mandated Macrogen Europe, Amsterdam, Netherlands.

Sequences were aligned manually using the programme BioEdit version 7.2.6.1 (Hall 1999). The alignment was made straight forward. The COI Barcode sequences obtained were matched to public sequences in the BOLD database (Ratnasingham and Hebert 2007), based on sequence similarity of at least 95%. For analysing the data via the Neighbour-joining method and Kimura 2-parameter model (Kimura 1980), we used MEGA version 7.0.26 (Kumar et al. 2016) with bootstrap replicates of 1000 (Felsenstein 1985).

### *Wolbachia* screening

For a screening over all collected species, at least one specimen was tested for the presence of *Wolbachia*-infection. From the two species *E. submarginalis* and *O. flexuosellus*, at least 15 specimens per locality were tested. This number of 15 individuals results from GPower (Faul et al. 2007) calculating the essential quantity for exploring high effects within a population.

We tested extracted DNA for the presence of *Wolbachia*-infection with PCR using two primer combinations. An approximately 1000 bp fragment is expected by the pair of primers 16sf 5´-TTG TAG CCT GCT ATG GTA TAA CT-3´/16sr 5´GAA TAG GTA TGA TTT TCA TGT-3´ (O'Neill et al. 1992). An approximately 550 bp fragment is expected by the pair of primers wsp-81F 5´-TGG TCC AAT AAG TGA TGA AGA AAC-3´/wsp-691R 5´-AAA AAT TAA ACG CTA CTC CA-3´ (Zhou et al. 1998). For finding a suitable annealing temperature for the multi-template PCR, first we assembled a temperature gradient, which directed to an annealing temperature of 50ºC.

The PCR was performed for both primer pairs simultaneously in 20 µl reactions, containing 10 pmol of each primer, 10mM dNTPs, PCR buffer, 50mM MgCl_2_ and 1U taq DNA Polymerase (ampliTaq, Thermo Fisher Scientific). After an initial phase at 95ºC for 5 min, the temperature profile was 95ºC for 30 sec, 50ºC for 45 sec and 72ºC for 1 min for a total of 38 cycles. The final elongation temperature was 72ºC for 10 minutes, followed by a cooling phase at 8ºC. To ascertain the results, every PCR contained a positive sample and a negative sample as well. PCR reactions that produced ambiguous results were re-run.

PCR products were visualised on 1% agarose gel and GelRed as dye agent. Specimens tested positive for *Wolbachia*-infection were determined by referring to positive control in each PCR reaction. For sequencing the *Wolbachia* DNA by using the primer pairs mentioned above, we mandated Macrogen Europe, Amsterdam, Netherlands. The wsp sequences obtained were matched to public sequences in Genbank database, based on sequence similarity of at least 95%. Sequences have been analysed via the Wolbachia PubMLST Databases (Baldo et al. 2006) and will be publicly available in the Barcode of Life Data System (Ratnasingham and Hebert 2007) in conjunction with the project NZPyr. Access to data has been restricted until included barcode and nuclear sequences are published (Wöger et al. in press).

## Results

During the surveys, 41 pyraloid species were obtained by 1–10 individuals, seven species by 11–50 individuals, five species by 51–200 individuals, as well as three species by more than 200 individuals.

**Figure 1.**
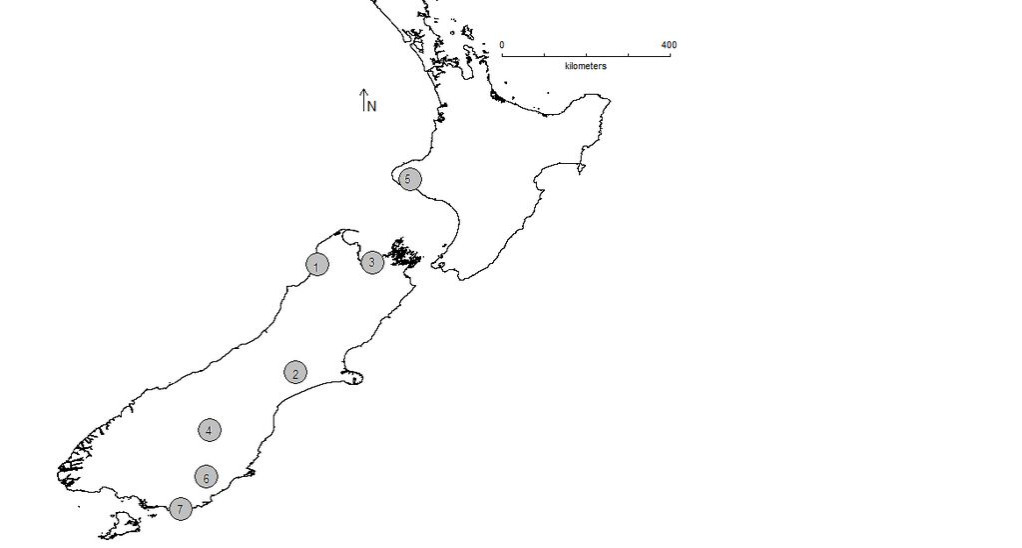
New Zealand map showing collection localities which are numbered as follows: 1: Karamea 2017, 2: Methven 2017 and Methven 2018, 3: Nelson 2017, 4: Cambrians 2018, 5: Taranaki Hollard Garden 2017, 6: Lawrence 2018, 7: Waikawa 2018

**Figure 2.**
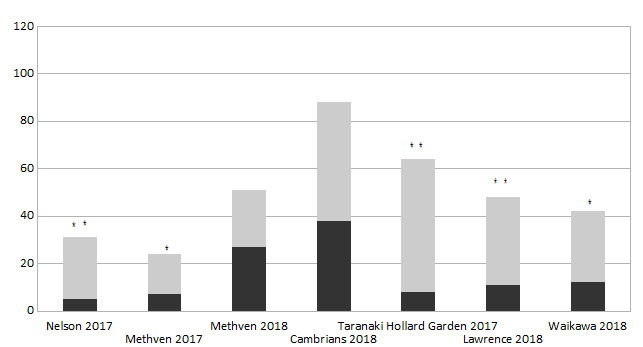
Sex ratio at different localities. Number of males (black) and females (grey) in *Orocrambus flexuosellus* . * samples with significant difference to an equal ratio of sexes with p ≤ 0.05, ** samples with significant difference to an equal ratio of sexes with p ≤ 0.001 (chi-square-test: χ 2 = 0.00011) .

**Figure 3.**
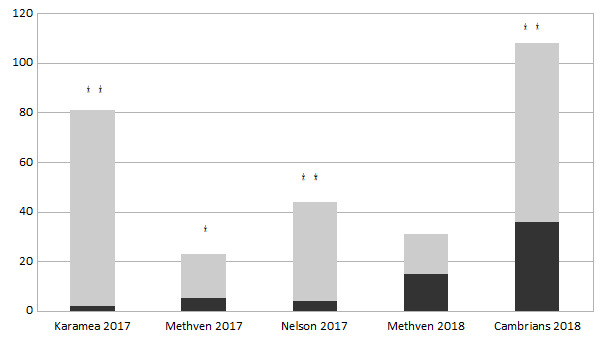
Sex ratio at different localities. Number of males (black) and females (grey) in *Eudonia submarginalis*. * samples with significant difference to an equal ratio of sexes with p ≤ 0.05, ** samples with significant difference to an equal ratio of sexes with p ≤ 0.001 (chi-square-test: χ 2 = 0.00011) .

**Figure 4.**
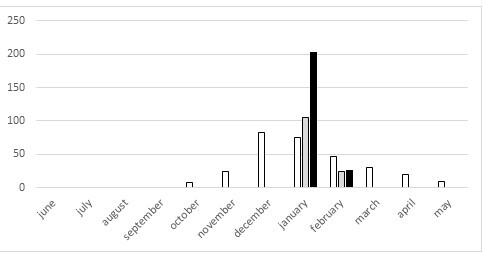
Timing and abundance of sampling of adults of *Orocrambus flexuosellus* in 2017 (grey) and 2018 (black) compared to long term monitoring data by Brian Patrick 1979–2014 (white).

**Figure 5.**
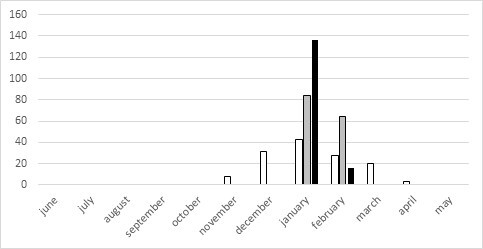
Timing and abundance of sampling of adults of *Eudonia submarginalis* in 2017 (grey) and 2018 (black) compared to long term monitoring data by Brian Patrick 1979–2014 (white) .

*Eudonia submarginalis* was found at seven localities, with more than 15 individuals at four localities, each on South Island (Figs 1, 3). *Orocrambus flexuosellus* was found at eight localities, with more than 15 individuals at six localities each, both on North and South Island (Figs 1, 2). The flight periods of *E. submarginalis* and *O. flexuosellus* are almost identical with a peak in January (Figs 4, 5).

### Sex ratio

The sex ratios are significantly (p ≤ 0.05) biased towards females in populations of *E. submarginalis* at four out of five localities, as well as of *O. flexuosellus* at five out of seven localities (Figs 2, 3).

### *Wolbachia* screening

We screened 56 pyraloid species for *Wolbachia*-infection. Specimens of eight species (14%) tested positive, of which six species belong to Scopariinae and two to Crambinae and Spilomelinae, respectively. Altogether, 13 males and 22 females tested *Wolbachia* positive (Table 1).

**Figure 6.**
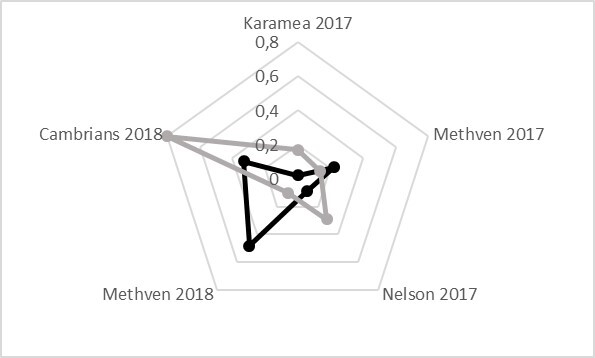
Relation of sex ratio and *Wolbachia* positive tested specimens in *E. submarginalis* at different localities. Sex ratio (black), *Wolbachia* positive tested specimens (percentage) (grey).

The more detailed screening of *E. submarginalis* and *O. flexuosellus* revealed a percentage of *Wolbachia* positive tested specimens in *E. submarginalis* of up to 80% depending on locality (N males tested = 18, infected = 8; N females tested = 38, infected = 14). No *Wolbachia* infection has been found in *O. flexuosellus* (N males tested = 23, females tested = 49 (Table 2). No relation between *Wolbachia*-infection and sex ratio has been found (Fig. 6).

The highest number of infected specimens of *E. submarginalis* is found at the Cambrians with 16 infected and four non-infected specimens, but this does not correlate with the strongest shift in sex ratio bias towards females which is found in Karamea. Furthermore, the percentage of *Wolbachia* positive tested specimens does not correlate with the sex ratio bias in general. For instance, there are 10% *Wolbachia* positive tested specimens of *E. submarginalis* at Methven in 2018 with no indication of sex ratio bias and 14.3% at Methven in 2017 with a sex ratio bias of 0.22 in favour of females (Table 2Fig. 6).

## Discussion

The data of our survey show sex ratio deviation towards females in *Eudonia submarginalis* and *Orocrambus flexuosellus* - two of the most common pyraloid moths in New Zealand - at some, but not all localities. Although both species are synchronous and syntopic, there are seasonal and species specific differences as well, for example, a sex ratio bias is found for both species in Methven in 2017, but not in 2018 and at the Cambrians for *E. submarginalis*, but not for *O. flexuosellus*.

Interpretation of records for protandry or protogyny is impossible, because the sampled pyraloid specimens represent only temporal fractions of the populations, which becomes evident when being compared with the data by Patrick (2014) (Figs 4, 5). Accordingly, there are long flight periods for *O. flexuosellus* from October to May, as well as for *S. submarginalis* from November to April. These data are combined from many years, as well as from many localities all over New Zealand, showing a Gaussian distribution of flight time for the two species. Indeed, data from extensive light trapping of moths, as well as rearing experiments, suggest the development of one generation for *E. submarginalis*, but two or more generations per year for *O. flexuosellus* (Cowley 1988, Gaskin 2010). An observation of the two species, at least at one locality over an entire season, would be necessary in order to investigate the local phenology with the number of generations per year, the life span of males and females, as well as possible shifts of the sex ratio bias over time.

The occurrence of *Wolbachia* in New Zealand insects has been discovered just very recently (Bridgeman et al. 2018). Here, *Wolbachia*-infection in New Zealand Lepidoptera is shown for the first time. Amongst the 56 tested pyraloid species, eight are positive, of which six belong to Scopariinae, as well as two to Crambinae and Spilomelinae, respectively. Thus, 14% of the investigated species bear a *Wolbachia*-infection. This is lower than the infection incidences of 45% to 90% in Lepidoptera reported by several authors (Tagami and Miura 2004, Ahmed et al. 2015, Ilinsky and Kosterin 2017). In our study, several non-infected species are represented by only a few individuals and we have studied only 22% of the pyraloid fauna from New Zealand so far. Thus, we cannot provide a complete and conclusive picture here.

Looking at several populations of *E. submarginalis*, the percentage of positively tested individuals varies from 10 to 80%. A similar wide range of infection rate is also reported for Japanese populations of *Zizina emelina* (Sakamoto et al. 2014) and *Colias erate poliographus* (Narita et al. 2009), the latter with 100% infected specimens in some populations.

Comparing the two most common pyraloid species *E. submarginalis* and *O. flexuosellus* with reference to a *Wolbachia* infection, only *E. submarginalis* tested positive and all its investigated populations show at least one infected specimen. Thus, *Wolbachia* infection may contribute to the sex ratio bias in *E. submarginalis*, but not in *O. flexuosellus.* In conclusion, there is no congruent pattern between unequal distribution of sexes and *Wolbachia*-infection.

## Figures and Tables

**Table 1.** Screening for *Wolbachia* amongst pyraloid species collected in 2017 and 2018.

**Family**	***species***	**Individuals total number**	**N Individuals tested for *Wolbachia***	**N Individuals tested positive for Wolbachia**
Acentropinae	*Argyra strophaea*	1	1	0
Acentropinae	*Hygraula nitens*	32	1	0
Crambinae	*Gadira acarella*	5	3	0
Crambinae	*Glaucocharis auriscriptella*	3	1	0
Crambinae	*Glaucocharis chrysochyta*	2	1	0
Crambinae	*Glaucocharis elaina*	2	1	0
Crambinae	*Glaucocharis interrupta*	1	1	0
Crambinae	*Glaucocharis lepidella*	5	3	0
Crambinae	*Glaucocharis selenaea*	4	1	0
Crambinae	*Orocrambus angustipennis*	3	2	0
Crambinae	*Orocrambus apicellus*	12	2	0
Crambinae	*Orocrambus creneus*	56	6	0
Crambinae	*Orocrambus enchephorus*	1	1	1
Crambinae	*Orocrambus flexuosellus*	358	72	0
Crambinae	*Orocrambus ordishi*	4	4	0
Crambinae	*Orocrambus ramosellus*	190	14	0
Crambinae	*Orocrambus vitellus*	242	16	0
Crambinae	*Orocrambus vulgaris*	51	8	0
Musotiminae	*Musotima nitidalis*	1	1	0
Phycitinae	*Crocydophora cinigarella*	1	1	0
Phycitinae	*Delogenes limodoxa*	1	1	0
Phycitinae	*Patagoniodes farinaria*	9	2	0
Pyraustinae	*Uresiphita ornitopteralis*	2	1	0
Pyraustinae	*Uresiphita polygonalis*	12	2	0
Scoparinae	*Antiscopa elaphra*	1	1	0
Scoparinae	*Eudonia aspidota*	3	1	0
Scoparinae	*Eudonia cataxesta*	3	1	0
Scoparinae	*Eudonia chlamydota*	5	2	1
Scoparinae	*Eudonia colpota*	4	1	0
Scoparinae	*Eudonia cymatias*	17	4	0
Scoparinae	*Eudonia cyptastis*	2	1	0
Scoparinae	*Eudonia dinodes*	2	2	2
Scoparinae	*Eudonia diphteralis*	3	3	0
Scoparinae	*Eudonia dochmia*	3	1	0
Scoparinae	*Eudonia feredayi*	6	1	0
Scoparinae	*Eudonia leptalea*	159	3	0
Scoparinae	*Eudonia manganeutis*	1	1	0
Scoparinae	*Eudonia minualis*	16	1	0
Scoparinae	*Eudonia minusculalis*	10	1	0
Scoparinae	*Eudonia octophora*	8	2	0
Scoparinae	*Eudonia philerga*	13	1	0
Scoparinae	*Eudonia rakaiensis*	20	8	1
Scoparinae	*Eudonia sabulosella*	126	4	0
Scoparinae	*Eudonia submarginalis*	300	60	23
Scoparinae	*Eudonia trivirgata*	1	1	0
Scoparinae	*Scoparia animosa*	2	1	0
Scoparinae	*Scoparia chalicodes*	10	6	3
Scoparinae	*Scoparia cyameuta*	1	1	0
Scoparinae	*Scoparia halopis*	9	1	0
Scoparinae	*Scoparia rotuella*	9	3	1
Scoparinae	*Scoparia* sp.	6	3	0
Scoparinae	*Scoparia ustimacula*	2	2	0
Spilomelinae	*Deana hybreasalis*	1	1	0
Spilomelinae	*Leucinodes cordalis*	1	1	0
Spilomelinae	*Mnesictena flavidalis*	5	4	3
Spilomelinae	*Mnesictena marmarina*	3	1	0

**Table 2.** *Wolbachia* screening between *E. submarginalis* and *O. flexuosellus*. Significant difference of sex ratio to an equal distribution with p ≤ 0.05 (chi-square-test : χ 2 = 0.00011) is given in bold.

**Locality (see Fig. 1)**	**sex ratio (N male / N total)** **(total number of collected individuals in brackets)**	**N individuals tested for *Wolbachia***	**N individuals tested positive for *Wolbachia* (in percent)**
***E. submarginalis***			
Karamea 2017 (1)	**0.02** (81)	12	2 (16.7)
Methven 2017 (2)	**0.22** (23)	7	1 (14.3)
Nelson 2017 (3)	**0.09** (44)	7	2 (28.6)
Methven 2018 (2)	0.48 (31)	10	1 (10.0)
Cambrians 2018 (4)	**0.33** (108)	20	16 (80.0)
**total**	**0.22 (287)**	**56**	**22 (39.3)**
***O. flexuosellus***			
Taranaki Hollard Garden 2017 (5)	**0.13** (65)	9	0 (0.0)
Nelson 2017 (3)	**0.16** (31)	9	0 (0.0)
Methven 2017 (2)	**0.29** (24)	9	0 (0.0)
Methven 2018 (2)	0.53 (51)	10	0 (0.0)
Cambrians 2018 (4)	0.43 (88)	9	0 (0.0)
Lawrence 2018 (6)	**0.23** (48)	9	0 (0.0)
Waikawa 2018 (7)	**0.29** (42)	8	0 (0.0)
**Total**	**031 (348)**	**72**	**0 (0.0)**

## Hosting institution

Senckenberg Museum of Zoology, Dresden, Germany
